# Omalizumab and mepolizumab in the landscape of biological therapy for severe asthma in children: how to choose?

**DOI:** 10.1186/s13052-019-0737-4

**Published:** 2019-11-28

**Authors:** Mattia Giovannini, Francesca Mori, Simona Barni, Maurizio de Martino, Elio Novembre

**Affiliations:** 1 Allergy Unit, Department of Pediatrics, Anna Meyer Children’s University Hospital, Viale Pieraccini 24, 50139 Florence, Italy; 2 Post-Graduate School of Pediatrics, Department of Health Sciences, Anna Meyer Children’s University Hospital, Viale Pieraccini 24, 50139 Florence, Italy; 3 Department of Health Sciences, Anna Meyer Children’s University Hospital, University of Florence, Viale Pieraccini 24, 50139 Florence, Italy

**Keywords:** omalizumab, mepolizumab, biological therapy, severe asthma, children

## Abstract

Severe asthma has a substantial epidemiological impact on children and biological treatments can be an option to take into account, as they target specific molecules and pathways involved in its pathogenesis. Modern medicine is continuously and progressively oriented towards tailored treatments designed specifically for the pathology patterns observed in individual patients and identified as endotypes with associated biomarkers. In this regard, biologic treatments in asthma are one of the best examples. Among the biological drugs currently available, omalizumab is the one with the greatest amount of data on efficacy and safety, and the one we have more real-life clinical experience with. However, mepolizumab will likely be accessible soon globally for clinical use. Moreover, research on biological drugs for the treatment of severe asthma is expanding rapidly, with some molecules currently used in adult patients that could be registered also for pediatric use and new molecules that could be available in the future. On the other hand, due to this potential abundance of therapeutic options, new criteria could become necessary to guide clinicians through an evidence-based choice between omalizumab and these new drugs. For the same reason, more data collected specifically from pediatric clinical trials are necessary. In this review we aim to analyze the factors that could help clinicians make their choice and to highlight the unmet need for a more evidence-based choice.

## Introduction

Asthma is a chronic protean respiratory disease usually marked by a chronic inflammation of the airways. It is also characterized by a clinical history of respiratory symptoms such as dyspnea, chest tightness, wheezing and cough. These symptoms may vary in time, in association with a variable limitation of expiratory flow which can resolve spontaneously or with therapy [1]. Although the prevalence of asthma varies according to reference age and country, the data taken from the International Study of Asthma and Allergies in Childhood (ISAAC) phase three suggest that asthma symptoms affect about 13.7% of children aged 13–14 and 11.6% of those aged 6–7 worldwide [2]. Such figures require considerable economic and human resources both by the healthcare system and the patients’ families [2].

There is no agreement on the definition of severe asthma. As a matter of fact, different options can be found in the scientific literature. The international European Respiratory Society/American Thoracic Society (ERS/ATS) guidelines proposed to define the severity of asthma by the extent of the treatment carried out in order to gain control of the disease [3]. Asthma is therefore defined as “severe” if, during the previous year, it required treatments with high doses of inhaled corticosteroids (ICS) in association with long-acting β2-agonist or anti-leukotriene or theophylline – level 4 of the Global Initiative for Asthma (GINA) guidelines. It is also defined as “severe” if it required treatments with systemic corticosteroid, as stated in the same guidelines – level 5, for a time period ≥50% of the previous year in order to be acceptably controlled. Finally, the same definition applies whenever asthma cannot be controlled even after these therapies [3]. From an epidemiological point of view, severe asthma is estimated to affect 0.5% of the general pediatric population and 4.5% of pediatric patients with asthma [4]. When facing a case of severe asthma, it is important to reconsider and confirm the diagnosis so as to exclude alternative pathological conditions that could be included in differential diagnosis and, hence, need to be treated differently [3]. Distinguishing between severe asthma and uncontrolled asthma is also very important. Although a concomitance between them cannot generally be excluded, uncontrolled asthma can frequently be caused by inadequate access to health resources, psycho-social factors, comorbidities (such as obesity, gastro-esophageal reflux, rhino-sinusitis etc.), precipitating factors (such as exposure to smoke, irritants, allergens etc.) and by inadequate or inappropriate treatment techniques [5, 6]. Finally, it can occur if patients fail to adhere to their treatment plan. In case of suspected severe asthma, it is therefore necessary to consider, exclude or handle each of these elements individually, providing the patient with the necessary time for their clinical condition to improve. In case of insufficient or inadequate control of severe asthma despite all the measures taken, it is necessary to consider different treatments than the traditional ones, including the use of biological drugs. This kind of treatment must be performed in a third-level pediatric pneumology or allergology center with experience in the field.

Biological drugs can act selectively on some specific molecular pathways by blocking them. Moreover, they can work on specific pathogenic mechanisms underlying a pathological process. In reference to asthma, as early as in 2007, biological drugs were defined as “magic bullets in search of their targets” [7], which may be immunoglobulin E (IgE) or even some important interleukins involved in the pathogenesis of this clinical condition. In any case, biological drugs can target specific molecules and pathways involved in asthma pathogenesis [8].

### Monoclonal antibody anti-IgE: omalizumab

Omalizumab is a humanized monoclonal antibody produced by recombinant DNA techniques. More specifically, it is an immunoglobulin G1 (IgG1) antibody which is able to bind the free circulating IgE (anti-IgE mAb) [9–11]. (Table 1) This biological drug has a series of molecular effects that justify its effectiveness from a clinical point of view. In particular, omalizumab can reduce the level of circulating IgE by binding to the IgE constant Cε3 region, averting any interaction between free IgE and high, low affinity IgE receptors – respectively Fc epsilon RI receptor (FcεRI) and Fc epsilon RII receptor (FcεRII) on basophils, mast cells and other cells. This prevents any release of inflammatory agents, in association with a FcεRI down-regulation expression on basophils and mast cells [12]. In addition, it has been shown that omalizumab can also reduce the in-vivo expression of FcεRI on dendritic cells, a factor that can lead to a reduction in the allergens presentation to T cells and, consequently, to a decrease in the T helper 2 (TH2)-mediated allergic pathway activity [13]. Thanks to all these effects, omalizumab can down-regulate the production of mediators that are responsible for allergic inflammation by reducing the activation of mast cells and eosinophils [14, 15]. This biological drug is administered subcutaneously, with a dosage and a frequency (every 2 or 4 weeks) set consistently with a nomogram whose fundamental parameters are the total serum IgE level (30–1500 kU/L) and the weight of the individual patient [16]. This drug is usually administered in a hospital setting and patients must be monitored after the drug administration. However, some geographical differences among countries due to specific national policies exist, and omalizumab may also be administered by a caregiver, approved by a pediatric allergist or a pulmonologist, with an appropriate training.

**Table 1:** Treatments with biological drugs currently being approved for severe asthma with their target, age of registration, effects and relevant reference studies

Drug	Target	Age of registration	Effects	References
omalizumab	anti-IgE mAb	≥ 6 years (EMA) ≥ 6 years (FDA)	↓ asthma exacerbations ↓ asthma hospitalizations ↑ asthma control ↓ oral corticosteroids ↑ quality of life	[16] [17] [18] [19] [20] [21] [22] [23] [24]
mepolizumab	anti-IL-5 mAb	≥ 12 years (EMA) ≥ 6 years (FDA)	↓ asthma exacerbations ↑ asthma control ↓ systemic corticosteroids ↑ pulmonary function	[33] [34] [35] [36] [37] [38]
reslizumab	anti-IL-5 mAb	≥ 18 years (EMA) ≥ 18 years (FDA)	↓ asthma exacerbations ↑ asthma control ↑ pulmonary function ↑ quality of life	[41] [42] [43] [44]
benralizumab	anti-IL-5Rα mAb	≥ 18 years (EMA) ≥ 12 years (FDA)	↓ asthma exacerbations ↑ asthma control ↓ oral corticosteroids	[45] [46] [47] [48] [49] [50]
dupilumab	anti-IL-4Rα mAb	≥ 12 years (EMA) ≥ 12 years (FDA)	↓ asthma exacerbations ↑ pulmonary function	[51] [52] [53] [54] [55]

EMA = European Medicines Agency; FDA = Food and Drug Administration; IgE = immunoglobulin E; IL-4Rα = interleukin-4 receptor alfa; IL-5 = interleukin-5; IL-5Rα = interleukin-5 receptor alfa; mAb = monoclonal antibody

Omalizumab is registered by the Food and Drug Administration (FDA) and the European Medicines Agency (EMA) [16, 17]. It is indicated as an add-on therapy for 6-year or older children with moderate to severe persistent asthma, in association with positive skin prick test or specific serum IgE to a perennial aeroallergen and asthma sign/symptoms that cannot be controlled with inhaled corticosteroids and a long-acting inhaled β2-agonist. It is also designed for children with documented severe asthma exacerbations, frequent daytime symptoms or night-time awakenings and, if aged 12 or above, reduced lung function (forced expiratory volume in the first second, FEV1, < 80%) [16]. Many studies show the safety and efficacy of omalizumab [16–24], which have been specifically demonstrated in pediatric patients through clinical trials or real-life experiences published in the literature [19, 21, 22, 24]. Apart from the specific clinical indications for which it is prescribed, in the scientific literature there is much evidence showing that this biological drug has also proved effective in the treatment of seasonal asthmatic exacerbations during spring and autumn [25, 26], in patients with total IgE values > 2000 kU/L [27] and even in the treatment of intrinsic severe asthma [28]. Besides, omalizumab has proved to be capable of reducing the anatomopathological alterations induced at a bronchial level by asthma in adults [29]. It is important to underline that if patients do not clinically respond within 16 weeks from the start of therapy, e.g. showing improvement in terms of disease signs/symptoms or drugs use reduction, it is unlikely that a continuation of treatment with omalizumab will result in a positive response [3]. Therefore, it is reasonable to re-evaluate them at this time interval to decide whether or not to continue with the omalizumab therapy. The main adverse effects of omalizumab on patients aged 12 years and above undergoing asthma treatments include headache and injection site reactions such as redness, swelling, pain and itching (observed in 1 to 10 patients out of 100). In patients aged 6–12, they are headache and fever, as observed in more than 1 out of 10 patients [16, 17]. Furthermore, a recent systematic review has shown that omalizumab has a good safety profile and a good tolerability. In the analysis carried out by the authors, this biological drug showed no substantial differences, compared to placebo, in terms of adverse or serious adverse effects [19]. Even if the vast majority of adverse reactions are represented by mild reactions manageable with a pharmacological treatment of the specific signs and symptoms, severe reactions such as a systemic one or anaphylaxis are sporadic. In these cases, a proper therapy should be given to the patient: intramuscular adrenaline is the most important drug in case of anaphylaxis, and the administration of omalizumab should be interrupted according to a benefit/risk balance principle. There is evidence that patients with a high risk of helminths infestations are slightly more exposed under omalizumab treatment. This could be explained considering the role that IgE plays in the immune response against parasites. The discontinuation of the drug administration should be taken into account in patients not responding to proper anti-helminth therapies [16]. One of the main problems in omalizumab treatments – albeit common to all treatments with biological drugs – is represented by its direct as well as indirect costs related to the inevitable use of public and family health resources to carry out the periodic follow-up. Uncertainty about the optimal therapy duration is another issue. Some evidence in the literature seems to indicate a direct proportionality between the duration of the therapy and the increase in positive effects, also in the long term. This highlights the need to continue with the treatment for at least a year, in case of clinical response [30, 31].

### Monoclonal antibody anti-IL-5: mepolizumab

Eosinophils and their chemical mediators play an important role in airways inflammation caused by asthma. Interleukin-5 (IL-5) is a fundamental cytokine for the maturation, activation, proliferation and survival of eosinophils. Mepolizumab is a humanized monoclonal antibody belonging to the IgG1 subclass. It can bind IL-5 (anti-IL-5 mAb) and prevent its interaction with interleukin-5 receptor alfa (IL-5Rα) [32]. (Table 1) This biological drug is administered subcutaneously every month at a dose of 40 mg (children aged 6–11 years) - 100 mg (children aged ≥12 years and adults) [33]. It is registered for use in children aged 12 or above by EMA and in those aged 6 or above by FDA. It is indicated as an add-on therapeutic option in patients with severe refractory eosinophilic asthma [33, 34]. Many studies in the scientific literature have demonstrated the safety and efficacy of mepolizumab in patients with a blood eosinophils count > 150 cells/μL or > 300 cells/μL in the previous year [33–38]. Specific pediatric data on these topics come from clinical trials published in the literature such as Dose Ranging, Efficacy, and Safety with Mepolizumab in Severe Asthma (DREAM) [35], Mepolizumab Treatment in Patients with Severe Eosinophilic Asthma (MENSA) [36] enrolling asthmatic adults and children aged ≥12 years or Steroid Reduction with Mepolizumab Study (SIRIUS) [37] enrolling adult patients and pediatric patients aged ≥16 years. The most common adverse effect of mepolizumab is headache, which may affect more than 1 out of 10 patients. Reactions at the injection site and backache are also common, as they can occur in up to 1 out of 10 patients [33, 34]. As previously described for omalizumab, adverse reactions to mepolizumab can range from mild – definitely the most common – to severe. They need to be approached with a specific pharmacological treatment. The latter are rare and the choice to interrupt the administration of the drug is based on a benefit/risk balance principle. Mepolizumab and other anti-IL-5 drugs are not to be used in patients with parasitic infestations, given the fundamental role that this cytokine plays in the immune response against such microorganisms. For this reason, in patients potentially eligible for mepolizumab therapy, investigations must be carried out to exclude a parasitic infestation. In case of a positive response, it is necessary to eliminate the infestation before beginning with the treatment [33]. Also, therapies with mepolizumab have the same issues as those with omalizumab in terms of costs and finding the optimal duration of treatment. The only difference is that, since this biological drug has been approved more recently than omalizumab, more time and further research will be required to find a solution to these problems. It is interesting to note that the National Institute for Health and Care Excellence (NICE) in the United Kingdom suggests re-evaluating patients on mepolizumab-based therapy after 12 months to verify if the frequency of asthmatic exacerbations has been reduced by at least 50% [39, 40]. This could be a parameter to decide whether or not to continue the therapy with this biological drug, even if the optimal therapy duration is still uncertain.

### Other present and future therapeutic options

Apart from omalizumab and mepolizumab, other innovative biological drugs targeting different molecules – approved (Table 1) or under investigation – can be considered. Reslizumab is a monoclonal antibody which, like mepolizumab, can bind IL-5 (anti-IL-5 mAb) and therefore prevent its interaction with IL-5Rα. It has been registered by EMA and FDA as a therapeutic add-on option in adult patients with severe eosinophilic asthma [41–44]. Therefore, reslizumab must currently be considered as off-label in pediatric age. Benralizumab is a monoclonal antibody capable of binding IL-5Rα (anti-IL-5Rα mAb). For this reason, it can inhibit the IL-5 pathway. It has been registered as a therapeutic add-on option in patients with severe eosinophilic asthma for adults by EMA and for 12-year or older children by FDA [45–50]. Interleukin-4 (IL-4) has a key role in the activation of TH2-mediated allergic inflammation. Dupilumab is a monoclonal antibody capable of binding interleukin-4 receptor alfa (IL-4Rα) (anti-IL-4Rα mAb). In addition to its use in patients with moderate to severe atopic dermatitis who are candidates for systemic therapy, dupilumab has been registered by EMA and FDA as a therapeutic add-on option in patients with severe eosinophilic asthma for children aged 12 or above [51–55]. For this reason, dupilumab may represent an interesting option for the treatment of both clinical conditions if they arise together in the same patient. Research on biological drugs for the treatment of severe asthma is rapidly expanding thanks to the experimentation of new molecules which, in the coming years, could further enrich the therapeutic options available for pediatric allergists and pulmonologists [56]. Interleukin-13 (IL-13) has a key role in the activation of TH2-mediated allergic inflammation as well. Therefore, this cytokine is another potential therapeutic target. Molecules capable of inhibiting its pathways, such as tralokinumab or lebrikizumab – monoclonal antibodies which can bind IL-13 (anti-IL-13 mAb), are currently under investigation [57, 58]. For the future of anti-IgE therapy, it is necessary to point out that a new monoclonal antibody – ligelizumab (anti-IgE-mAb) – is currently being tested, as it has shown an affinity for human IgE approximately fifty times higher than omalizumab, with a nine-fold increase in the suppressive power of circulating free IgE levels [59]. From a clinical point of view, ligelizumab seems to be more effective than omalizumab in controlling the asthmatic response to inhalation allergens [60]. In addition to anti-IgE monoclonal antibodies, a new category of drugs is currently being tested. They are called anti-CεmX-mAb because they act on CemX domain on membrane-bound IgE, and they have a molecular target located further upstream than the direct blocking of circulating IgE. This category of monoclonal antibodies operates with an alternative mechanism on the IgE-mediated allergic inflammatory pathway. They bind to the IgE expressed on the membrane of IgE-switched B lymphoblasts, causing lysis and preventing the allergen-mediated generation of IgE-producing plasma cells [61]. These biological drugs do not bind to free IgE and therefore their action does not depend on serum IgE levels in treated patients. However, it should be pointed out that so far, from a clinical point of view, these drugs have not given satisfactory results. For example, quilizumab, a drug belonging to this category, has not had any appreciable clinical benefit in adults with allergic asthma not controlled by standard therapy [62].

## Discussion

Biological drugs represent an option in case of insufficient or inadequate control of severe asthma after therapy administration according to level 4 and 5 of the GINA guidelines. Among the biological drugs currently available, omalizumab is today the one with the greatest amount of data about efficacy and safety, and the one we have more real-life clinical experience with. However, research on biological drugs for the treatment of severe asthma is expanding rapidly, with new molecules currently being registered for pediatric patients such as mepolizumab (≥ 6-years by EMA and ≥ 12 years by FDA), benralizumab (≥ 12 years by FDA) and dupilumab (≥ 12 years by EMA and FDA). (Table 1) Still, not all of these biological treatments are already available in all countries. There is a heterogeneous distribution that depends on national factors such as economy and domestic policies. With the exception of omalizumab, such heterogeneity is limiting the practical experience with these new biological drugs to the research studies. Other molecules currently used in adult patients, such as reslizumab, could be registered for pediatric use. Moreover, new molecules could be available in the future. On the other hand, this potential abundance of therapeutic options also requires new head-to-head comparative studies between the latter and omalizumab. These studies should be carried out on patients with severe asthma who are eligible for more than one treatment, so the clinician could make an evidence-based choice about the drug to administer. This unmet need seems to be particularly important for mepolizumab, as it has the greatest amount of data in the scientific literature – after omalizumab – and it will probably be available soon worldwide for clinical use. In case of clinically-relevant total IgE increase (30–1500 kU/L) and allergic sensitization proven through skin tests or serum specific IgE, it is reasonable to consider the use of omalizumab as an option. In the presence of biomarkers for eosinophilic inflammation and TH2 inflammatory endotype such as eosinophilia (with a of blood eosinophils count > 150 cells/μL or > 300 cells/μL in the previous year), instead, the use of mepolizumab could be taken into account. However, sometimes these criteria could overlap and, although there are some data about an indirect comparison in the scientific literature, further research on a head-to-head comparison between these two drugs remains necessary.

Magnan et al. [63] collected data from two double-blind placebo-controlled studies MENSA [36] and SIRIUS [37], carrying out a post hoc analysis to estimate the safety and efficacy of mepolizumab in patients suffering from severe eosinophilic asthma who had previously undergone omalizumab treatment. The study showed a comparable rate of asthma exacerbations: in MENSA, annual exacerbations rate – rate ratio (RR) = 0.43, 95% confidence interval (95% CI) = 0.21–0.89 – and RR 0.53, 95% CI 0.41–0.70 versus placebo in the group with or without prior omalizumab treatment respectively; in SIRIUS, annual exacerbations rate RR = 0.67, 95% CI 0.36–1.23 and RR = 0.71, 95% CI 0.45–1.14 versus placebo in the group with or without prior omalizumab treatment respectively. A comparable decrease in the use of oral corticosteroids (OCS) was also noticed: in SIRIUS, ≥50% reduction from baseline – odds ratio (OR) = 2.53, 95% CI = 0.69–9.32 – and OR = 2.33, 95% CI = 0.93–5.80 versus placebo in the group with or without prior omalizumab treatment respectively. The study also showed similar rates of asthma control assessed through the Asthma Control Questionnaire-5 (ACQ-5): in MENSA, change from baseline − 0.87, 95% CI -1.46, − 0.28 and − 0.38, 95% CI -0.56,-0.21 versus placebo in the group with or without prior omalizumab treatment respectively; in SIRIUS, change from baseline − 0.44, 95% CI -1.05, 0.18 and − 0.55, 95% CI -0.98,-0.13 versus placebo in the group with or without prior omalizumab treatment respectively. A comparable quality of life was observed, too, assessed with the St. George’s Respiratory Questionnaire (SGRQ): in MENSA, change from baseline −12.1, 95% CI -23.5, −0.7 and −6.2, 95% CI -9.1, −33.3 versus placebo in the group with or without prior omalizumab treatment respectively; in SIRIUS, change from baseline −3.4, 95% CI −11.9,5.0 and −7.2, 95% CI -12.9, −1.4 in the group with or without prior omalizumab treatment respectively. Finally, no substantial differences in the number of any treatment-related adverse events were observed: in MENSA, 26% in the mepolizumab group versus 24% in the placebo group and 18% in the mepolizumab group versus 15% in the placebo group in patients with or without prior omalizumab treatment respectively; in SIRIUS, 23% in the mepolizumab group versus 30% in the placebo group and 30% in the mepolizumab group versus 16% in the placebo group in patients with or without prior omalizumab treatment respectively. Therefore, the authors concluded that these patients responded well to mepolizumab, even if they had previously received omalizumab. However, the mean age of the patients who underwent mepolizumab therapy in the two trials was respectively 48.2 years (range 13–76 years) and 50.5 years (range 12–82 years) for the group with or without prior administration of omalizumab. From the age distribution of the patients analyzed, it is clear how the vast majority of the patients studied was adult. Nonetheless, more information on the degree of response to mepolizumab after a treatment with omalizumab will be available, also in pediatric age, as soon as the final results of the Omalizumab to Mepolizumab Switch Study in Severe Eosinophilic Asthma Patients (NCT02654145) [64] are published. This is an open-label study enrolling children aged 12 and above as well as adults, in which patients with severe eosinophilic asthma who were receiving omalizumab without an optimal response were enrolled to switch to mepolizumab.

Cockle et al. [65] performed a systematic literature review and an indirect treatment comparison (Bayesian framework) in order to compare tolerability and effectiveness between omalizumab and mepolizumab as add-on therapies in severe asthma. The vast majority of the patients included in the studies analyzed were adults. This work analyzed two different populations: patients eligible for both drugs (overlap population) and patients eligible for only one drug (trial population). In the first population (overlap), a better trend for mepolizumab was noticed, but the study did not show any statistically-relevant difference between the two drugs in the rate of clinically-notable exacerbations (estimated median RR 0.66; 95% credible interval 0.37–1.19), in exacerbations leading to hospitalization (estimated median RR 0.19; 95% credible interval 0.02–2.32) or in treatment adverse events (estimated median RR 0.79; 95% credible interval 0.31–1.91). In the second population (trial), the study showed a statistically-significant difference in the rate of clinically notable exacerbations for mepolizumab (estimated median RR 0.63; 95% credible interval 0.45–0.89), but not in the rate of exacerbations leading to hospitalization (estimated median RR 0.58; 95% credible interval 0.16–2.13) or treatment adverse events (estimated median RR 0.79; 95% credible interval 0.44–1.40). However, due to the heterogeneity of the trials analyzed, this kind of analysis cannot be fully reliable. Therefore, the authors concluded that the tolerability characteristics of the two drugs did not differ significantly and that mepolizumab appeared to be at least as effective as omalizumab.

Nachef et al. [66] performed a network meta-analysis of the published literature and an efficacy comparison between omalizumab and mepolizumab in the treatment of severe asthma. The weighted mean age of the patients who underwent therapy with omalizumab or mepolizumab was 42.4 or 47.3 years respectively. The authors found out that the two drugs are equally effective. There were no significant data showing noteworthy differences in the Asthma Control Questionnaire (mean difference -0.02; 95% CI - 0.53,0.50 which favored mepolizumab), Asthma Quality of Life Questionnaire (mean difference -0.38; 95% CI -0.55,-0.21, with *p *< 0.0001 which favored omalizumab but did not reach the minimal value for a clinical impact equal to 0.5), FEV1 (mean difference 9.3 ml; 95% CI -67.7,86.3 which favored mepolizumab) and peak expiratory flow rate (PEFR) (mean difference 6.24 L/min; 95% CI -6.46,18.9 which favored mepolizumab). Due to the high heterogeneity of the criteria used to make such choice in the patient populations studied, the authors were not able to give any conclusive recommendations about the choice between omalizumab or mepolizumab.

A head-to-head comparison between omalizumab and mepolizumab will be available as soon as we get the results of the Study on Magnitude and Prediction of Response to Omalizumab and Mepolizumab in Adult Severe Asthma (PREDICTUMAB, NCT03476109) [67]. In this study, severe asthma patients who were eligible to receive both mepolizumab and omalizumab were randomized to decide the first treatment to start with. Then, according to their clinical response, the treatment was prolonged or the patients switched to the other drug. Such trial was performed on adult patients, but data concerning pediatric patients are necessary, too. Moreover, it is worth noting that most of the first clinical trials about biological drugs for the treatment of severe asthma included few children or did not distinguish between the outcomes obtained in children and in adults. For this reason, it is important to get more information about their efficacy in pediatric age, especially for newer molecules. Data regarding long-term safety of these drugs are necessary, too, especially in children. Pharmaco-economy elements will also be essential to choose the biological drug for severe asthma in pediatric age, to optimize the cost/benefit ratio among all the therapeutic options and to make their use sustainable in different national health systems. At the moment, mepolizumab is more expensive than omalizumab, also because it is a newer molecule. However, this could change in the future, and the choice between these two drugs will have to be based on well-designed cost-benefit studies. Such works should take into account the savings from the use of the two drugs in comparison to the drugs’ costs. In the precision-medicine era, trying to endotype asthma-specific characteristics through the analysis of some elements and the study of certain biomarkers could be very useful. It could help identify tailored treatments with biological drugs in cases of severe asthma [8, 69]. This could reduce the number of ineffective treatments and, therefore, costs. For this reason, further research on asthma endotypes is necessary to choose the most adequate drug for each case, but also to find new potential drug targets [68].

## Conclusions

Biological drugs can be an option in case of severe asthma [70–72]. Among the biological drugs currently available, today omalizumab is the one with the biggest amount of data on efficacy and safety, and the one we have more real-life clinical experience with [73]. However, research on biological drugs for the treatment of severe asthma is expanding rapidly, with some molecules currently used in adult patients that could be registered also for pediatric use. Moreover, even more molecules could be available in the future. On the other hand, this potential abundance of therapeutic options also calls for new head-to-head comparative studies between these new drugs and omalizumab. Such studies should be carried out on patients eligible for more than one treatment in order to guide clinicians through an evidence-based choice of a specific drug. More data about the efficacy of these drugs in pediatric patients are needed, especially for newer molecules [74]. Information about their optimal therapy duration and long-term safety is also necessary, particularly in children [75].

Modern medicine is constantly and progressively oriented towards tailored treatments designed specifically for the pathology patterns observed in individual patients and identified as endotypes with associated biomarkers. Biologic treatments in asthma are one of the best examples in this field. Further research on asthma endotypes remains necessary to choose the most adequate drug for each case and also to find new potential drug targets [68].

## Abbreviations

95% CI: 95% confidence interval
ACQ-5: Asthma Control Questionnaire-5
ATS: American Thoracic Society
DREAM: Dose Ranging, Efficacy, and Safety with Mepolizumab in Severe Asthma
EMA: European Medicines Agency
ERS: European Respiratory Society
FcεRI: Fc epsilon RI receptor
FcεRII: Fc epsilon RII receptor
FDA: Food and Drug Administration
FEV1: forced expiratory volume in the first second
GINA: Global Initiative for Asthma
ICS: inhaled corticosteroids
IgE: immunoglobulin E
IgG1: immunoglobulin G1
IL-13: interleukin-13
IL-4: interleukin-4
IL-4Rα: interleukin-4 receptor alfa
IL-5: interleukin-5
IL-5Rα: interleukin-5 receptor alfa
ISAAC: International Study of Asthma and Allergies in Childhood
mAb: monoclonal antibody
MENSA: Mepolizumab Treatment in Patients with Severe Eosinophilic Asthma
NICE: National Institute for Health and Care Excellence
OCS: oral corticosteroids
OR: odds ratio
PEFR: peak expiratory flow rate
RR: rate ratio
SGRQ: St. George’s Respiratory Questionnaire
SIRIUS: Steroid Reduction with Mepolizumab Study
TH2: T helper 2
